# Risk of falls in people with chronic kidney disease and related factors*

**DOI:** 10.1590/1518-8345.3911.3289

**Published:** 2020-06-08

**Authors:** Thaís Carrera de Carvalho, Ariane Polidoro Dini

**Affiliations:** 1 Universidade Estadual de Campinas, Faculdade de Enfermagem, Campinas, SP, Brazil.

**Keywords:** Renal Insufficiency, Chronic, Renal Dialysis, Accidental Falls, Patient Safety, Nursing, Fall, Insuficiência Renal Crônica, Diálise Renal, Acidentes por Quedas, Segurança do Paciente, Enfermagem, Queda, Insuficiencia Renal Crónica, Diálisis Renal, Accidentes Por Caídas, Seguridad del Paciente, Enfermería, Caer

## Abstract

**Objective:**

to identify the risk and prevalence of falls in the last year in chronic renal failure patients on hemodialysis; to associate the risk of falls with the fear of falling and socio-demographic-clinical variables.

**Method:**

association study. 131 individuals participated in the study. The Morse Falls Scale, the Fall Efficacy Scale and the Tilburg Frailty Indicator were used. The data were analyzed by linear regression, the level of significance adopted was 0.05.

**Results:**

97.7% were at risk for falls and 37.4% had at least one fall *per* year, with a mean of 2.02. Extreme concern about falling was presented by women, patients with less education, amputees, and frail individuals. Diabetes, as a comorbidity, and people with difficulty or need for assistance for ambulance showed a significant increase in the occurrence of falls.

**Conclusion:**

high risk and high prevalence of falls were found in hemodialysis patients, greater in those with diabetes or mobility limitations. Fear of falling was identified especially in women and in people with less education. These findings challenge the role of preventing falls, both in hemodialysis sessions and in the adoption of strategies for activities of daily living that involve patients and their families.

## Abstract (pt)

**Objetivo:**

identificar o risco e a prevalência de quedas no último ano em doentes renais crônicos em hemodiálise; associar o risco de queda com o medo de cair e variáveis sociodemográfico-clínicas.

**Método:**

estudo de associação. Participaram do estudo 131 indivíduos. Foram utilizados a Escala de Quedas de Morse, o Fall Efficacy Scale e o Tilburg Frailty Indicator. Os dados foram analisados por regressão linear, o nível de significância adotado foi de 0,05.

**Resultados:**

97,7% apresentaram risco para quedas e 37,4% apresentaram pelo menos uma queda ao ano, com média de 2,02. Apresentaram extrema preocupação em cair: as mulheres, os pacientes com menor nível de escolaridade, os amputados e os frágeis. A diabetes, enquanto comorbidade, e pessoas com dificuldade ou necessidade de auxílio para a deambulação apresentaram aumento significante quanto à ocorrência de quedas.

**Conclusão:**

constatou-se alto risco e alta prevalência de quedas nos pacientes em hemodiálise, maiores naqueles com diabetes ou com limitações na mobilidade. O medo de cair foi identificado especialmente nas mulheres e pessoas com menor escolaridade. Esses achados desafiam a protagonizar a prevenção de quedas, tanto nas sessões de hemodiálise, quanto na adoção de estratégias para as atividades de vida diária que envolvam pacientes e seus familiares.

## Abstract (es)

**Objetivo:**

identificar el riesgo y la prevalencia de caídas en el último año en pacientes con insuficiencia renal crónica en hemodiálisis; asociar el riesgo de caer con el miedo a caer y las variables sociodemográficas-clínicas.

**Método:**

estudio de asociación. 131 personas participaron en el estudio. Se utilizaron la Escala de Caídas de Morse, la Fall Efficacy Scale y el Tilburg Frailty Indicator. Los datos fueron analizados por regresión lineal, el nivel de significancia adoptado fue de 0,05.

**Resultados:**

el 97,7% presentó riesgo de caídas y el 37,4% tenía al menos una caída por año, con un promedio de 2,02. Presentan extremada preocupación por las caídas: mujeres, pacientes con menos educación, y personas amputadas y frágiles. La diabetes, como comorbilidad, y las personas con dificultad o necesidad de asistencia para caminar mostraron un aumento significativo en la cantidad de caídas.

**Conclusión:**

se encontraron alto riesgo y alta prevalencia de caídas en pacientes en hemodiálisis, mayor en aquellos con diabetes o limitaciones de movilidad. El miedo a caer se identificó especialmente en mujeres y personas con menos educación. Estos hallazgos desafían el papel de la prevención de caídas, tanto en las sesiones de hemodiálisis como en la adopción de estrategias para las actividades de la vida diaria que involucran a los pacientes y a sus familiares.

## Introduction

The 2019 Global Kidney Health Atlas points out that 10% of the world population is affected by chronic kidney disease (CKD) consisting of kidney damage and irreversible loss of kidney function, present for more than three months, in need of dialysis treatment^(^ 1 ^-^ 2 ^)^.

CKD and hemodialysis are responsible for physical and emotional limitations with an important negative impact on the quality of life of people affected by this pathology, interfering in the performance of activities of daily living and restricting the social interaction of the individual, in addition to being associated with several comorbidities especially in elderly^(^ 1 ^-^ 3 ^)^.

Comorbidities related to CKD can cause functional limitations, low cardio-respiratory fitness, fatigue, disturbances of mineral metabolism, which lead to bone mineral disease, and can ultimately act as a risk factor for accidents due to falls^(^ 1 ^-^ 4 ^)^, which are present in greater number in chronic renal patients undergoing dialysis treatment^(^ 5 ^-^ 7 ^)^.

Patients on hemodialysis are at a higher risk of falls, ranging from 13% to 25%, compared to the general population^(^ 6 ^,^ 8 ^-^ 9 ^)^. The rate of falls after hemodialysis is significantly higher compared to pre-hemodialysis, revealing a negative effect of hemodialysis on postural stability^(^ 6 ^)^.

Falls in chronic renal patients undergoing hemodialysis are also associated with polypharmacy, frailty, advanced age and a previous history of falls^(^ 10 ^)^. However, there are still few studies that deal with this subject exactly.

Falls are defined by the World Health Organization as inadvertently falling to the ground or at another lower level^(^ 11 ^)^.

Falls can be classified as: accidental, when caused by environmental factors (such as water on the floor for example) or by loss of the patient’s balance that correspond to 14% of falls in general, anticipated physiological falls, which occur in patients classified with fall risk, that is, patients who have more than one episode of previous fall, weak or impaired pace and correspond to 78% of falls and unanticipated physiological falls, that is, those that cannot be predicted and can be associated with physiological causes such as fainting, pathological hip fractures, and correspond to 8% of falls^(^ 8 ^-^ 9 ^,^ 11 ^)^.

Another possibility of classifying falls may also be related to complications such as: without complications, minor complications (all other complications), major complications (such as fractures) and death^(^ 8 ^)^.

In individuals with chronic kidney disease on hemodialysis (HD) falls can be classified as to temporality: fall on a day that does not perform HD, fall before the HD session or after the HD session^(^ 8 ^)^.

The study of factors related to falls in patients undergoing hemodialysis makes it possible to identify prevention and safety promotion strategies for individuals with chronic kidney disease. Thus, the objectives of this study are to identify the risk and prevalence of falls in the last year in people with chronic kidney disease undergoing hemodialysis and to associate the risk of falling with the fear of falling and socio-demographic-clinical variables.

## Method

This is a quantitative, descriptive and association study. It was carried out in a nephrology service in the city of São João da Boa Vista, in the inland of the state of São Paulo, which assists patients from the Brazilian Public Health System (*Sistema* Único *de Saúde*, SUS) and from private health plans. The study site serves approximately 230 patients on hemodialysis.

The sample size was calculated considering the score obtained using the instrument that assesses the risk of falls as a dependent variable and as a set of 13 variables as independent variables: fear of falling, gender, age, education, marital status, comorbidities, time in hemodialysis, medication use, limb amputation, difficulty walking, aid in ambulance, bone mineral disease and frailty.

To perform the sample calculation, the G*Power 3.1.9.2 software was used. The level of significance was set at 5%, test power of 80%, and medium degree effect size (0.15)^(^ 12 ^)^. Thus, the sample consisted of 131 chronic renal patients undergoing hemodialysis.

To be a participant in the research, the inclusion criteria were chronic kidney patients aged 18 years old or over on Renal Replacement Therapy, in hemodialysis modality, for more than six months.

The exclusion criteria were patients who were not self-, halo- and chrono-psychically oriented, patients on peritoneal dialysis, due to their low representativeness and monthly attendance at the service.

The research project was submitted to the Research Ethics Committee (*Comitê de* Ética *em Pesquisa*, CEP) of the University linked to the researchers. After approval opinion number 2874412/2018, the eligible patients were invited to participate in the study, being informed about the purpose and preservation of their identities. Then, after reading and signing the Free and Informed Consent Form, they became participants in the study.

Data collections were performed between November 2018 and January 2019, in the hemodialysis room, during the procedure, with the participant accommodated in the armchair. The first author of the study applied the questionnaires and the collection time varied between 10 to 20 minutes *per* participant.

Four instruments were used, one of socio-demographic and clinical characterization of the participants, built specifically for this study and the other three validated for Brazilian culture, the Morse Fall Scale^(^ 13 ^)^, the Fall Efficacy Scale (FEI-I - Brazil)^(^ 14 ^)^, and the Tilburg Frailty Indicator (TFI)^(^ 15 ^)^.

The Morse Scale has the objective of identifying people at risk of anticipated physiological falls. It consists of six questions, with scores between 0 and 30, with the total sum varying between 0 and 125. The final score to determine the risk of falling is defined as ≤24 (no risk of falling), 25 to 50 (low risk of falling) and ≥51 (high risk of falling)^(^ 13 ^-^ 14 ^)^.

TheFall Efficacy Scale(FEI - I - Brazil) was used to measure the fear of falling. The instrument addresses 16 daily activities at different levels, including external activities and social participation, with the total score varying from 16 (no concern) to 64 (extreme concern)^(^ 15 ^)^.

The TFI was used to measure frailty. Although the instrument is made up of two parts, only part B, which identifies the weakness itself, was used in this study. The assessment of frailty is made up of 15 objective questions, self-reported, distributed in three domains: physical, psychological and social. The final score ranges from 0 to 15 points, with the highest score meaning a higher level of frailty, or alternatively scores higher than five points indicate that the individual is frail^(^ 16 ^)^.

Data such as medications, comorbidities, time on hemodialysis, among others, were collected from the patients’ medical records, by the first author of the study.

To study the associations between qualitative variables, the Chi-square test was applied, and for cases where the assumptions of the Chi-square test were not met, Fisher’s exact test was applied. For the comparisons involving a qualitative variable and a quantitative variable, the Mann-Whitney non-parametric test or the unpaired Student t test was applied, according to the data distribution^(^ 12 ^)^.

In a second stage of the analysis, multiple Poisson regression models were constructed with robust variance. In the results, the estimates obtained from the prevalence ratio were presented, as well as their respective confidence intervals and p-values^(^ 12 ^)^. For all the analyses, a level of significance equal to 5% was considered.

All data were tabulated in an electronic spreadsheet and analyzed using the statistical SAS software, version 9.4.

## Results

Of the 131 people who made up the sample, 52.6% were men and 47.3% women. The mean age of the participants was 56.09 years old. 55.7% of the interviewees declared having a partner, being married or in a stable relationship; 44.2% declared themselves single, widowed or divorced. The mean number of years of schooling was 7.79.

In the analysis of the comorbidities of the participants, 60.8% had an isolated diagnosis of arterial hypertension; 28% had hypertension and diabetes mellitus simultaneously; and 8.3% had an isolated diagnosis of diabetes mellitus. Other comorbidities recorded to a lesser extent by the other individuals included: autoimmune disease, polycystic kidneys, heart disease, glomerulonephritis, among others.

In the period of one year, the occurrence of at least one fall was reported by 37.4% of the participants; the average number of falls was 2.02; and, a single participant reported 10 falls in the period.

Table 1 shows the relationship between the number of falls and the socio-demographic variables and comorbidities experienced by patients on hemodialysis.

**Table 1:** Occurrence of falls in chronic renal patients. São João da Boa Vista, SP, Brazil, 2018-2019 (n* = 131)

Variable	Fall last year				p^†^
	No		Yes		
	n	%	n	%	
Gender					0.0820^‡^
Male	48	69.5	21	30.4	
Female	34	54.8	28	45.1	
Marital status					0.3273^‡^
No partner	39	67.2	19	32.7	
Has a partner	43	58.9	30	41.1	
Systemic Arterial Hypertension					0.1283^‡^
No	17	51.5	16	48.4	
Yes	65	66.3	33	33.6	
Diabetes Mellitus					0.0067^‡^
No	58	71.6	23	28.4	
Yes	24	48.0	26	52.0	
Limb amputation					0.5356^§^
No	74	61.6	46	38.3	
Yes	8	72.7	3	27.2	
Difficulty walking					0.0314^‡^
No	51	70.8	21	29.1	
Yes	31	52.5	28	47.4	
Walking aid device					0.0025^‡^
No	76	67.8	36	32.1	
Yes	6	31.5	13	68.4	
Bone Mineral Disease					0.6483^‡^
No	66	61.6	41	38.3	
Yes	16	66.6	8	33.3	
Tilburg					0.4834^‡^
Not frail	47	65.2	25	34.7	
Frail	35	59.3	24	40.6	

* n = Number of patients;

† p = Value;

‡ p = Value obtained through the Chi-Square test;

§ p = Value obtained through Fisher’s exact test

According to the data collected in the application of the instrument, 93.8% of the participants with chronic kidney disease had some risk of falls, with 37.4% presenting a high risk for falls and 60.3% presenting a low risk for falls.

Table 2 shows the association between the risk of falling and the socio-demographic and clinical variables.

**Table 2:** Risk of falls in chronic renal failure patients. São João da Boa Vista, SP, Brazil, 2018-2019 (n* = 131)

	Morse Scale				p^†^
	No risk/Low risk		High risk		
	n	%	n	%	
Gender					0.6193^‡^
Male	43	62.3	26	37.6	
Female	36	58.0	26	41.9	
Marital status					0.4671^‡^
No partner	37	63.7	21	36.2	
Has a partner	42	57.5	31	42.4	
Systemic Arterial Hypertension					0.4343^‡^
No	18	54.5	15	45.4	
Yes	61	62.2	37	37.7	
Diabetes Mellitus					0.0237^‡^
No	55	67.9	26	32.1	
Yes	24	48.0	26	52.0	
Limb amputation					1.0000^§^
No	72	60.0	48	40.0	
Yes	7	63.6	4	36.3	
Difficulty walking					0.0001^‡^
No	54	75.0	18	25.0	
Yes	25	42.3	34	57.6	
Walking aid device					0.0002^‡^
No	75	66.9	37	33.0	
Yes	4	21.0	15	78.9	
Bone Mineral Disease					0.4809^‡^
No	63	58.8	44	41.1	
Yes	16	66.6	8	33.3	
Tilburg					0.3544^‡^
Not frail	46	63.8	26	36.1	
Frail	33	55.9	26	44.0	

* n = Number of participants;

† p = Value ;

‡ p = Value per Chi-square test;

§ p = Value by Fisher’s exact test

In the analysis of the daily activities, such as outdoor activities and social participation, in the assessment of fear of falling men were less concerned in relation to women (p<0.05). Non-amputees and non-frail individuals are also less concerned about falling when compared to amputees and frail individuals, respectively.

Diabetic and hypertensive patients did not show statistically significant differences regarding the concern with falling in relation to non-diabetic and non-hypertensive patients.

Schooling also influenced the fear of falling, so that the group of people with an average education of 8.27 years had little or no fear of falling, while the group of people with an average education of 6.04 years showed extreme fear of falling (p-value = 0.0492/Mann-Whitney test). The other associations between fear of falling and the socio-demographic and clinical variables are shown in Table 3.

**Table 3:** Fear of falling in chronic kidney patients. São João da Boa Vista, SP, Brazil, 2018-2019 (n* = 131)

Variable	FES^†^ (fear of falling)				p^‡^
	No/Little		Very/Extremely		
	n	%	N	%	
Gender					0.0040^§^
Male	61	88.4	8	11.5	
Female	42	67.7	20	32.2	
Marital status					0.8648^§^
No partner	46	79.3	12	20.6	
Has a partner	57	78.0	16	21.9	
Systemic Arterial Hypertension					0.6421^§^
No	25	75.7	8	24.2	
Yes	78	79.5	20	20.4	
Diabetes Mellitus					0.1461^§^
No	67	82.7	14	17.2	
Yes	36	72.0	14	28.0	
Limb amputation					0.7010^ǁ^
No	95	79.1	25	20.8	
Yes	8	72.7	3	27.2	
Difficulty walking					<0.0001^§^
No	67	93.0	5	6.9	
Yes	36	61.0	23	38.9	
Walking aid device					0.2395^ǁ^
No	90	80.3	22	19.6	
Yes	13	68.4	6	31.5	
Bone Mineral Disease					0.1138^§^
No	87	81.3	20	18.6	
Yes	16	66.6	8	33.3	
Tilburg					<0.0001^§^
Not frail	66	91.6	6	8.3	
Frail	37	62.7	22	37.2	

* n = Number of participants;

† FES = Fall Efficacy Scale;

‡ p = p-value;

§ p = Value by Chi-square test;

ǁ p = Value by Fisher’s exact test

As shown in Table 4, the reasons for the prevalence of falls were estimated for “the occurrence of falls in the last year”, “high risk of falling” and “Very/Extremely concerned about falling” and the variables, which showed statistically significant differences for this study.

**Table 4:** Prevalence of falls in chronic kidney patients. São João da Boa Vista, SP, Brazil, 2018-2019 (n* = 131)

Independent variables	PR^†^	CI^‡^ (95%)		p^§^
		Lower limit	Upper limit	
Fall in the last year				
Gender (ref^ǁ^ = Male)	1.61	1.03	2.50	0.0358
DM^¶^ (ref = No)	1.93	1.19	3.13	0.0075
Walking aid device (ref = No)	2.32	1.35	4.00	0.0023
High risk of fall				
Difficulty walking (ref = No)	1.88	1.14	3.10	0.0131
Walking aid device (ref = No)	1.97	1.26	3.08	0.0032
“Very/Extremely concerned about falling”				
Schooling	0.94	0.88	0.99	0.0399
Difficulty walking (ref = No)	4.74	1.85	12.16	0.0012
Tilburg (ref = Not frail)	2.47	1.03	5.94	0.0435

* n = Number of participants;

† PR= Prevalence ratio;

‡ IC = Confidence interval;

§ Poisson regression;

ǁ ref = reference;

¶ DM = Diabetes mellitus

## Discussion

It was found that the individuals with chronic kidney disease on hemodialysis have a high prevalence of falls, so that patients with diabetes, with difficulty or need for assistance in ambulance have a higher prevalence of falls.

In the assessment of the participants, regarding the risk of falls, it was identified that those who experienced the same clinical variables related to the high prevalence of falls, that is, people with diabetes, difficulty or need for assistance for ambulance had higher scores on the Morse scale, which represents the high risk for falls^(^ 13 ^)^.

When comparing groups of chronic kidney patients who also have diabetes mellitus, chronic kidney patients who have difficulty ambulance and those who use orthoses showed a statistically significant difference in terms of the greater number of falls in relation to the other patients who did not have these characteristics.

Hypertension as a comorbidity did not influence the results so that there was no statistically significant difference, with respect to the risk and the occurrence of falls between non-hypertensive participants and normotensive participants.

In assessing the fear of falling by the scale of fall effectiveness, women had higher scores in daily activities such as outdoor activities and social participation, which denotes extreme concern (extreme fear) of falling^(^ 14 ^)^.

Another variable that interfered with the fear of falling was the number of years of schooling, so that patients with higher education have little or no fear of falling, while patients with less education have extreme fear of falling.

The comparison of prevalence and risk of falls among the participants classified as frail in the assessment of the physical, psychological and social domains^(^ 15 ^)^ in relation to patients not classified as frail found no significant differences. This finding also diverged from the literature, where frailty, described as a highly vulnerable state for adverse clinical outcomes, has been investigated as a risk factor for falls in people with kidney diseases^(^ 16 ^)^.

No statistically significant differences were identified regarding the number of falls in the last year, neither the risk nor the occurrence of falls between male participants and female participants. These findings differ from those found in a systematic review that identified in other studies that women have significant numbers for falls^(^ 16 ^)^.

Among people with a simultaneous diagnosis of bone mineral disease, there was no statistically significant difference in the number and risk of falls in relation to participants without this comorbidity. The findings are in contrast to studies that found an increased risk of falls in people with bone mineral disease, regardless of the degree of bone remodeling, in addition to greater impairment of the physical aspects of quality of life^(^ 17 ^)^. The association with quality of life with these findings was not the scope of this study, constituting an important gap regarding the importance of other studies that assume the quality of life of people with chronic kidney disease and the occurrence of falls.

No significant differences were identified in the risk and prevalence of falls between amputated and non-amputated participants, a possible interpretation of this indifference would be that individuals who have used prostheses for long periods are adapted after the rehabilitation period.

Likewise, there was no significant difference between the occurrence and the risk of falls, between people who had a partner or not. In face of the contemporaneity of sharing the responsibilities of caring for people with chronic pathologies with close friends or family, as well as the growing presence of informal caregivers^(^ 18 ^-^ 20 ^)^ possibly strategies developed on a daily basis by these caregivers, may justify the absence of a relationship between the risk or number of falls and living with spouses.

In this theme, with regard to functional independence, that is, the ability to perform activities without assistance, individuals with chronic kidney disease are dependent on walking to go up and down stairs, with 10.2% of patients needing help to perform this activity; and, in relation to mobility, 18.4% of patients have some dependence^(^ 21 ^)^.

Given the impact that this fear of falling can have on quality of life by limiting activities of daily living and social activities to women and people with less education, specific support strategies for these people with chronic kidney disease arise, which can be performed by family, caregivers, nursing and interdisciplinary staff.

The contribution of this study carried out with the participation of people with chronic kidney disease on hemodialysis was to identify the high prevalence of falls in this very specific population, which should be treated with international standards of care and prevention of falls, both during hemodialysis sessions, and in the adoption of strategies to prevent falls in the activities of daily living of these people.

A study is suggested that contemplates the involvement of family members in the care and quality of life of people with chronic kidney disease and the occurrence of falls.

The limitations of the study were that it was conducted in a single nephrology center, which limits the generalization of the results, and the absence of a specific instrument to assess the risk of falls of chronic renal patients on hemodialysis.

## Conclusion

There was a high risk and high prevalence of falls in individuals with chronic kidney disease undergoing hemodialysis. The extreme fear of falling has been identified especially in women. In addition, related factors such as diabetes, difficulty or need for ambulance assistance increased the occurrences of falls.

The analysis of the risk and the prevalence of falls, as well as other conditions that interfere in the quality of life of people with chronic kidney disease, challenge the specialized nursing to update itself and deal with standards of excellence in care and prevention of falls, both during hemodialysis sessions, as well as the adoption of educational and fall prevention strategies in the activities of daily living of these individuals and their families.
